# Improvement in the Post-Processing of Wave Buoy Data Driven by the Needs of a National Coast and Sea Monitoring Agency

**DOI:** 10.3390/s23125371

**Published:** 2023-06-06

**Authors:** Giovanni Battista Rossi, Gabriele Nardone, Giulio Settanta, Marco Picone, Marta Berardengo, Francesco Crenna

**Affiliations:** 1 Department of Mechanical, Energy, Management and Transportation Engineering, University of Genova, Via Opera Pia 15A, 16145 Genova, Italy; marta.berardengo@unige.it (M.B.); francesco.crenna@unige.it (F.C.); 2 ISPRA, Istituto Superiore per la Protezione e la Ricerca Ambientale, Via V. Brancati 48, 00144 Rome, Italy; gabriele.nardone@isprambiente.it (G.N.); giulio.settanta@isprambiente.it (G.S.); marco.picone@isprambiente.it (M.P.)

**Keywords:** sea waves, wave buoys, directional spectrum, directional spreading function

## Abstract

Technological development in terms of the power requirement for data acquisition and processing opens new perspectives in the field of environmental monitoring. Near real-time data flow about the sea condition and a possible direct interface with applications and services devoted to marine weather networks would have a significant impact on several aspects, such as, for example, safety and efficiency. In this scenario, the needs of buoy networks have been analyzed, and the estimation of directional wave spectra from buoys’ data has been deeply investigated. Two methods have been implemented, namely the truncated Fourier series and the weighted truncated Fourier series, and they have been tested by both simulated and real experimental data, representative of typical Mediterranean Sea conditions. From simulation, the second method proved to be more efficient. From the application to real case studies, it emerged that it works effectively in real conditions, as confirmed by parallel meteorological observations. The estimation of the main propagation direction was possible with a small uncertainty of a few degrees, yet the method exhibits a limited directional resolution, which suggests the need for undertaking further studies, briefly addressed in the conclusions.

## 1. Introduction: Post-Processing Needs for a National Wave Buoy Network

### 1.1. Historical

Today, the study of the sea wave energy spectrum from in situ data is still a major challenge. Despite the modeling approach being increasingly consolidated and effective, this challenge also applies to the determination of extreme events, both in regional and local areas [1,2,3]. For this reason, among recent decades, the most industrialized countries have equipped themselves with offshore monitoring networks, consisting of directional wave buoys capable of acquiring the spectral parameters of wave motion. These instruments measure the spectral features of sea waves at a given point. The upgrade to an extended buoy network allows for the monitoring of the sea state along entire stretches of coast.

The Italian national wave buoy network, Rete Ondametrica Nazionale (RON), managed by the Italian Institute for Environmental Protection and Research, Istituto Superiore per la Protezione e la Ricerca Ambientale (ISPRA), has reached more than thirty years of activity, despite some significant interruptions. It is one of the oldest networks in the world devoted to the monitoring of the physical state of the sea and is based on directional wave buoys. In fact, the RON was first set up in the late 1980s (1 July 1989), with eight wave buoys featuring heave-pitch-roll directional wave sensors to measure wave directional spectra [4]. Wave energy was also measured and normalized in engineering units as translation measurements with respect to the three axes, including compass correction. In normal operation, the attribution time was based on a synoptic hour, with every survey of the wave motion beginning about 20 min before this time. In the following years, the network was upgraded and improved with further spherical directional buoys, based on the translational principle, to form a maximum overall network of fifteen measuring stations [5]. In the first phase of the monitoring network, the data acquisition took place only at eight synoptic hours (acquisition in three-hour time steps), and the complete spectral analysis was available only when the waves exceeded certain thresholds. In the following phases, instead, considerable efforts on analysis procedures were made in order to obtain statistical data from physical historical series in real time (acquisition on a thirty-minute base), processed directly onboard. An important aspect of a buoy network setup is the trade-off between the power needs of the buoys and their performance in data acquisition and processing. Currently, the RON buoys have a single annual intervention, for ordinary maintenance and battery recharging.

From 2009, to expand the monitoring potential of the network, which depends on the characteristics of each buoy, meteorological wave buoys have been deployed, larger in size and displacement than previous models to allow the acquisition of the main meteorological parameters [6]. For the high frequencies of wave motion, the accuracy of inertial wave measurements decreases with increasing buoyancy and weight of the buoy, regardless of the platform’s own transfer function. In the last decade, ISPRA has achieved the use of buoys of about 700 kg buoyancy with a height from the sea level of about 2.5 m. While maintaining good accuracy and excellent nautical qualities in terms of reserve of thrust and safety for navigation, they leave space for an additional payload, which was used to install meteorological instruments.

In order to ensure the continuity of the observed time series while pursuing the constant updating and improvement of the measuring devices, requirements for the accuracy and precision of the equipment were adopted. Today, ISPRA, which has started the complete upgrade of the RON network with a different type of buoys, intends to update the national standards for processing wave spectra for homogeneity of wave measurements. To accomplish this goal, it’s necessary to reprocess the observed signals on shore to produce reliable spectra for the Italian seas. The procedure includes reevaluations of the raw data and comparisons among different analysis tools in order to reduce statistical uncertainties in the estimation of wave parameters.

To comply with its mandate of environmental data open access, since 2016, ISPRA has been releasing its data from the RON monitoring network in Linked Open Data (LOD) format. The advantage of LOD resides in its standardized format and machine readability. Data can be accessed at portal http://dati.isprambiente.it/ (accessed on 29 May 2023) through specific SPARQL queries [7]. (SPARQL is the standard language for such queries.) After passing a dedicated filtering procedure, synthetic parameters calculated from RON data are published in the format of text files on a monthly base.

The quality of these data is considered of primary importance because it must guarantee the accuracy of the monitoring. For this reason, ISPRA applies a specific data quality control procedure that is based on international guidelines, both in real time and delayed [8]. The real-time data quality control procedure is developed as follows: A nominal 30-min data slice is filtered on the signal with a seventh-order low-pass Butterworth filter with a frequency cutoff of 0.6 Hz to avoid aliasing errors. The sample is transmitted every 0.78 s as a 128-bit sequence of 1536 samples. The receiver decodes the 128-bit unit, locating and correcting the most significant impairments. Furthermore, the absolute value and the direction of the magnetic field are calculated. Since both values must remain almost stable in normal conditions, a comparison is made with average values based on the 256 previously valid samples to perform the quality test. Evaluated data values are accepted if the mean absolute value of the earth’s magnetic field distortion is below 10% and if the direction of the terrestrial magnetic field has a mean distortion of less than 5.5°. For validation purposes, the measured heave value is compared with the expected one obtained from the interpolation of the previous and following values to remove anomalous values from the series [9].

### 1.2. Currents Needs

Presently, automated procedures run onboard and produce synthetic parameters based on two analyses, in the time and frequency domains, respectively. The related software is designed by the buoy manufacturer, and the encapsulated algorithms are able to solve the full non-linear equations that define the buoy motions, starting from the raw data acquired by the onboard instruments.

The frequency analysis, used to extract the non-directional and directional spectra, is performed inside a selected frequency range, which is optimized for oceanic data.

**Figure 1:**
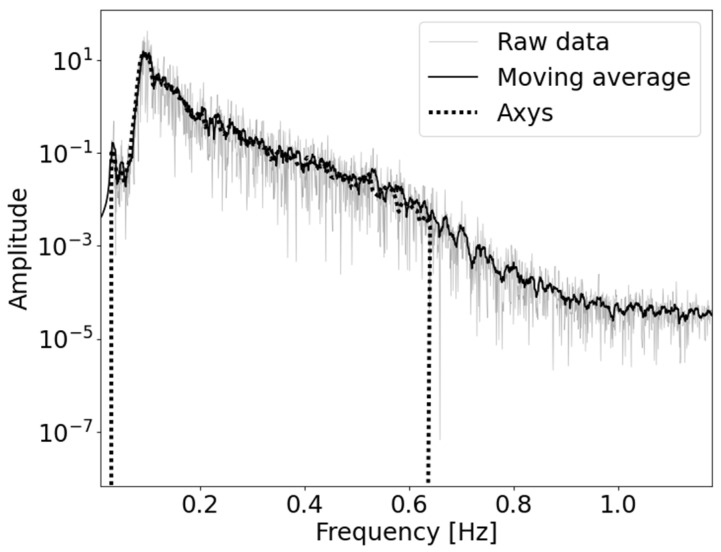
Frequency amplitude spectrum, integrated over one example run. “Raw data” consists of the raw frequency spectrum without any pre-processing procedure. A moving average on the raw data is also reported to smooth the statistical fluctuations. The spectrum generated by the buoy onboard software is labelled as “Axys”.

The features of a closed sea like the Mediterranean Basin, however, can differ significantly from those of an open ocean. It is therefore reasonable to establish an independent procedure with no (or different) initial assumptions about the sea conditions. Such a procedure can benefit from the long historical series acquired by RON, consisting of more than three decades of statistics. In Figure 1, an example of the frequency amplitude spectrum of a typical Axys Watchkeeper^TM^ buoy run is reported, before and after processing by the Axys company’s onboard software and from the raw data. Here, the motion along the vertical axis is considered. As can be seen, the filters operating onboard consider a frequency range of the whole spectrum. By means of a separated and open procedure, possible non-standard features in the buoy record can be addressed, as well as specific patterns of the sea area around the buoy.

### 1.3. Future Perspectives

Recent technological developments in terms of processing power today offer many opportunities for environmental monitoring networks. Moreover, the demand for an easily readable and portable data output illuminates the need for a standardized data format. The fast spread of IoT technologies will eventually add a new dimension for monitoring data requirements, to “move toward an integrated, multidisciplinary and multiscale system of systems, where heterogeneity should be exploited to deliver fit-for-purpose products that answer the diversity and complexity of the requirements from stakeholders and end-users” [10].

Within this framework, one of the potential improvements for ISPRA RON is the full automation of the current procedure for data acquisition, validation, LOD transformation, and publication, to be performed onboard the buoy itself. This would allow a near real-time data flow about the sea condition, leading to a possible direct interface with applications and services devoted to marine weather, such as decision support systems [11]. Through these tools, for example, it is possible to optimize navigation routes to avoid stormy sea areas or increase navigation safety to reduce the risk of accidents, predict the drift trajectories of a pollutant and also of a shipwrecked person, or even increase the safety and efficiency of offshore platforms and ports by managing the openings of these activities, by developing coastal flood warning models, etc.

At the same time, data quality must be at an optimal level, considering all the necessary adjustments for sea-specific conditions.

With minor adjustments, present technology already supports such an upgrade, which would result in a direct benefit for the population.

Major challenges are related to the estimation of directional wave spectra. Directional wave spectra (DWS) convey vital information for many applications, including wave modeling and the prediction of the interactions between waves and other matter. Even if the notion of DWS has been investigated for a long time, its actual implementation in a national climate monitoring system, in terms of instrumentation, data acquisition, and post processing, to produce reliable results, is still an object of investigation and improvement, as mentioned in the previous section [12,13,14].

Therefore, two methods for the estimation of DWS have been implemented and carefully tested by both simulated and real signals. Developing methods that are not license-protected is of key importance for a national wave buoy monitoring system in order to ensure the analysis stability across different buoy providers throughout the years. Some preliminary results were presented in Ref. [15]. This paper is organized as follows: Some general aspects about the modeling and the spectral estimation techniques are addressed in Section 2. The methods considered herein have been implemented and tested on both simulated and real data. Simulation tests (throughout the paper, the term “test” is used for simulation and the term “case study” for application to real data) include the synthesis of realizations from both discrete (deterministic) and continuous (stochastic) spectra, and results are shown in Section 3. Case studies include three representative sea state conditions, observed in the Mediterranean Sea, where some of the authors operate, including different wind directions and both mono- and bimodal spectra. These are also particularly important in view of the specificity of the Mediterranean Sea, as will be discussed later. Results are reported in Section 4 and discussed with respect to the corresponding general meteorological conditions. Lastly, conclusions are drawn and future development is addressed in Section 5.

## 2. Directional Wave Spectra

### 2.1. General Issues

Let the wave elevation be represented as the stationary random process z(t); then, its directional spectrum (power spectral density) can be factorized as

(1) $$S\left(f,\theta \right)=S_{zz}\left(f\right)D\left(f,\theta \right),$$

where $S_{zz}(f)$ is the non-directional elevation power spectral density (PSD) and D(f,θ), is a directional spreading function. The non-directional spectrum can be restored from the directional one by marginalization:

(2) $$S_{zz}\left(f\right)=\int_{0}^{2\pi }S\left(f,\theta \right)d\theta ,$$

and the directional spreading function, as a distribution, should be non-null, as follows:

(3) $$\forall f\forall \theta ,D\left(f,\theta \right)\geq 0,$$

and should satisfy the normalization condition:

(4) $$\int_{0}^{2\pi }D\left(f,\theta \right)d\theta =1.$$

To estimate the directional spectrum from data, a Fourier series expression is often assumed for the directional dispersion function, as operationally used by the National Data Buoy Center (NDBC) [16] and the Coastal Data Information Program (CDIP) at the Scripps Institution of Oceanography [17], which is

(5) $$D\left(f,\theta \right)=\frac{1}{2\pi }+\frac{1}{\pi }\sum_{n=1}^{\infty }\left[a_{n}\left(f\right)\cos n\theta +b_{n}\left(f\right)\sin n\theta \right]$$

This study focuses on analysis techniques based on the conventional Fourier series, postponing to another time the comparison with the other method traditionally used at the CDIP, which is based on the maximum entropy method (MEM) [18].

### 2.2. Implementation Aspects

The estimation of directional wave spectra from the data of wave buoys can be based either on heave-pitch-roll data [19,20,21,22] or on three-dimensional displacement data [13,23,24], depending on the kind of buoy employed. The former data are typically related to surface-following or pitch-and-roll buoys that follow wave slope and are typically disc-shaped. The latter are rather provided by wave-following or translational buoys, which follow water particles and are typically spherical-shaped [25]. In this study, displacement data were considered.

Before providing some details on the method used, let us briefly introduce some notation concerning, in particular, spectral analysis [26]. Let *u*(*t*) and *v*(*t*) be two generic stochastic ergodic random processes (this is a shorthand notation for u(ω,t) and v(ω,t), where ω is the sample index). Then, their cross-correlation function can be defined by

(6) $$R_{uv}\left(\tau \right)=E\left[u\left(t\right)v\left(t+\tau \right)\right].$$

If the integral of its absolute value exists, their cross-spectrum (CPSD: cross power spectral density) is the Fourier Transform of the cross-correlation, which is

(7) $$S_{uv}\left(f\right)=\int_{-\infty }^{+\infty }R_{uv}\left(\tau \right)exp\left(-j2\pi f\tau \right)d\tau ,$$

where *j* is the imaginary unit. In sea wave monitoring, one-sided spectra are usually considered because of their simpler physical interpretation. The one-sided cross spectrum $G_{uv}(f)$, where f varies only over (0,+∞), is defined by

(8) $$G_{uv}\left(f\right)=2S_{uv}\left(f\right)=C_{uv}\left(f\right)-jQ_{uv}\left(f\right),$$

where $C_{uv}(f)$ is called the coincident spectrum (co-spectrum) and $Q_{uv}(f)$ the quadrature spectrum (quad-spectrum).

In the present study, the methods of the truncated Fourier series (TFS) and the weighted truncated Fourier series (WTFS) were considered [27,28]. Let us now consider the estimation of the directional spectrum, based on three-dimensional displacement data. Let us then consider the following processes:

- x(t): displacement toward East,
- y(t): displacement toward North, and
- z(t): elevation (heave).

Then, the series terms in (5) are considered only up to the second order (n=2) and are provided by the following equations:

(9) $$a_{1}\left(f\right)=\frac{Q_{zx}\left(f\right)}{\sqrt{C_{zz}\left(f\right)\left(C_{xx}\left(f\right)+C_{yy}\left(f\right)\right)}},$$

(10) $$b_{1}\left(f\right)=\frac{Q_{zy}\left(f\right)}{\sqrt{C_{zz}\left(f\right)\left(C_{xx}\left(f\right)+C_{yy}\left(f\right)\right)}},$$

(11) $$a_{2}\left(f\right)=\frac{C_{xx}\left(f\right)-C_{yy}\left(f\right)}{C_{xx}\left(f\right)+C_{yy}\left(f\right)},$$

(12) $$b_{2}\left(f\right)=\frac{2C_{xy}\left(f\right)}{C_{xx}\left(f\right)+C_{yy}\left(f\right)}.$$

Therefore, in the TFS approach, the directional spreading function becomes [16,27]

(13) $$D\left(f,\theta \right)=\frac{1}{2\pi }+\frac{1}{\pi }\left(a_{1}\cos\theta +b_{1}\sin\theta +a_{2}\cos2\theta +b_{2}\sin2\theta \right).$$

Yet, in this formulation, negative values may appear. To avoid that, the weighted truncated Fourier series (WTFS) was proposed, which reads

(14) $$D\left(f,\theta \right)=\frac{1}{2\pi }+\frac{2}{3\pi }\left(a_{1}\cos\theta +b\sin\theta \right)+\frac{1}{6\pi }\left(a_{2}\cos2\theta +b_{2}\sin2\theta \right).$$

Both approaches will be considered and discussed in the following sections.

### 2.3. Spectrum Estimation Methods

The required spectra can be estimated in different ways [29,30,31]. In this study, Welch’s overlapped segment averaging (WOSA) approach has been adopted [32]. Testing is planned for other approaches in the ensuing development of this study.

In the WOSA method, the available data record of duration T is parsed in segments of duration $T_{0}$ with partial overlap. Each segment is pre-treated by tapering with a smooth window, w, to reduce the bias due to spectral leakage, and the square of the discrete Fourier transform is calculated for each of them. For historical reasons, these partial results are called (modified) periodograms, and the final estimate is obtained by averaging such periodograms. In this approach, tapering allows us to contain bias and averaging to reduce variance.

Let us then denote the series of measurements by $x_{i}=x(i∆t)$, where ∆*t* is the sampling interval, $i=1,\ldots N,T=N∆t,\;and\;T_{0}=N_{0}∆t$. Let $w_{1},\ldots ,w_{N_{0}}$ be a data taper, and then the modified periodogram for the *l*-th segment is

(15) $${\hat{S}}_{l}\left(f\right)=∆t{\left|\sum_{i=1}^{N_{0}}w_{i}x_{i+l-1}e^{-j2\pi fi∆t}\right|}^{2},$$

and the final estimate is given by

(16) $$\hat{S}\left(f\right)=\frac{1}{n}\sum_{k=0}^{n-1}{\hat{S}}_{km+1}\left(f\right),$$

where n is the number of segments and m is an integer-valued shift factor, satisfying $0<m\leq N_{0}$ and $m(n-1)=N-N_{0}$.

To optimize the result, proper choice of the analysis features is required. They include the kind of taper, the degree of overlap, and, especially, the duration of individual segments, $T_{0}$. In particular, the choice of $T_{0}$ greatly influences the quality of the result: it should be large enough to provide a good spectral resolution and small enough for reducing the variance, allowing us to average a greater number of segments. Criteria for proper choice of analysis parameters, for both unimodal and bimodal sea states, were developed and proposed in [29,30]. Here, a cosine (Hanning) taper was adopted, with a 50% overlap. This is the original setup proposed by Welch and has proven to be effective in many circumstances. It is also recommended in [16]. With this choice, in [29] it emerged that for a unimodal sea state, for a long duration (T=3600 s), a segment duration $T_{0}=120\;s$ proved to be optimal, while for a short duration (T=600 s), $T_{0}=80\;s$ was best. In the case of bimodal spectra, larger values are recommended, ranging from $T_{0}=100\;s$ in the case of short duration up to $T_{0}=240\;s$ for long duration [30]. In this study, for simulation, a duration of T=1320 s has been typically adopted, which is similar to the one occurring in typical monitoring systems, and $T_{0}=120\;s$ was chosen. Additional details on the choice of $T_{0}$ and on the corresponding expected performance will be given in Section 4.

## 3. Implementation and Testing of the Procedure

### 3.1. Testing on Discrete (Deterministic) Spectra

Both approaches to directional spectrum estimation, namely TFS and WTFS, have been implemented in a MatLab environment, with spectral estimation based on the WOSA method, and have been tested through signals obtained by simulation. Firstly, harmonic signals with few frequencies with different directions were considered, and then signals outcoming from continuous stochastic processes were examined.

**Figure 2:**
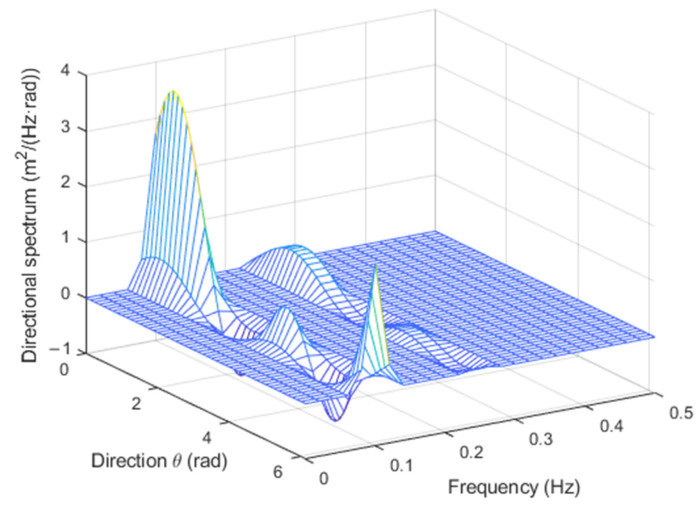
Directional spectrum for the test signal with the TFS approach.

**Figure 3:**
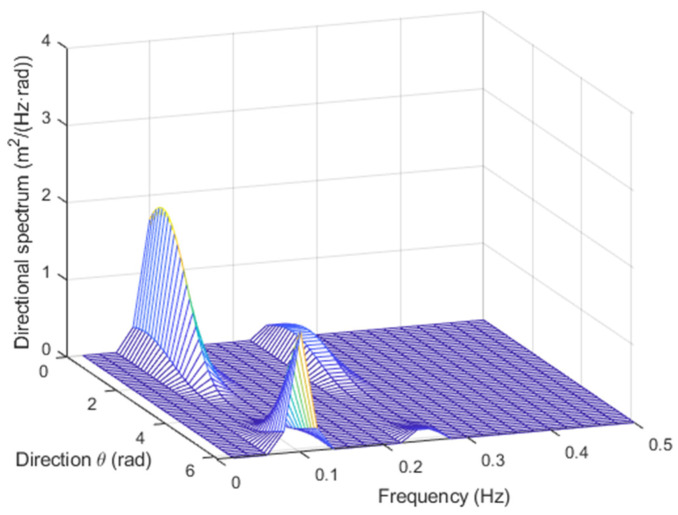
Directional spectrum for the test signal with the WTFS approach.

In the case of discrete (deterministic) spectra, we obtain the following:

(17) $$z\left(t\right)=\sum_{n=1}^{N}a_{n}\cos\left[-2\pi f_{n}t+\varphi_{n}\right],$$

(18) $$x\left(t\right)=\sum_{n=1}^{N}{-a}_{n}\cos\theta_{n}\sin\left[-2\pi f_{n}t+\varphi_{n}\right],$$

(19) $$y\left(t\right)=\sum_{n=1}^{N}{-a}_{n}\sin\theta_{n}\sin\left[-2\pi f_{n}t+\varphi_{n}\right],$$

with $a_{n}=H_{n}/2$, where $H_{n}$ is the *n*-th wave height, and $f_{n}=1/T_{n}$, where $T_{n}$ is the *n*-th wave period. For example, for N=2, $H_{1}=1$ m, $H_{2}=0.5$ m, $T_{1}=10$ s, $T_{2}=4$ s, $\theta_{1}=30{}^{\circ}$, and $\theta_{2}=60{}^{\circ}$, with the TFS approach, the directional spectrum in Figure 2 was obtained, and with the WTFS method, the one in Figure 3 was obtained.

In both cases, the directions of the two components are clearly detected. There are some border artifacts, close to the 360° region, that depend on the low-order approximation of the Fourier series in (5). However, they disappear if the result is plotted in the range ($\theta_{max}-180{}^{\circ},\theta_{max}+180{}^{\circ}$) rather than (0, 360°), as will be shown in the next subsection.

However, it appears that the former approach gives rise sometimes to negative values of the spectrum, which are physically meaningless.

This does not happen with the latter approach, which, on the other hand, has a lower spectral resolution, as can be noticed by the lower values of the peaks.

After considering these two aspects, the authors generally prefer and recommend the latter approach. Both, however, have been occasionally considered in the following and compared, when needed.

### 3.2. The Main Direction and the Directional Spread Function

At each frequency, the main direction can be estimated by

(20) $$\theta_{0}\left(f\right)=\mathrm{atan}\left(\frac{b_{1}\left(f\right)}{a_{1}\left(f\right)}\right),$$

where $a_{1}\left(f\right)$ and $b_{1}\left(f\right)$ are defined in (9) and (10), respectively. In the example above, the two directions were correctly identified. Furthermore, an overall direction can be estimated by weighted averaging, as follows.

Introducing the overall “power” of the elevation signal,

(21) $$P=\int_{0}^{f_{max}}S\left(f\right)df,$$

where $S(f)=S_{zz}(f)$, the average propagation direction can be estimated as

(22) $$\theta_{0}=\frac{1}{P}\int_{0}^{f_{max}}\theta_{0}\left(f\right)S\left(f\right)df.$$

**Figure 4:**
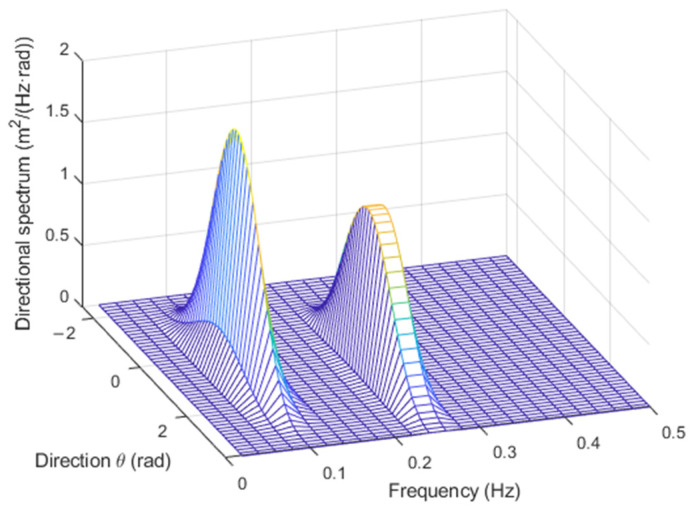
Directional spectrum centered in the main propagation direction.

In the example above, $\theta_{0}=36{}^{\circ}$ was obtained, which makes sense, since most of energy was in the component having direction at 30°. If the directional spectrum is plotted in the range (−144°, +216°), it appears as in Figure 4, where the effects have disappeared.

In much the same way, it is suggested to calculate the overall directional spreading function as follows:

(23) $$D\left(\theta \right)=\frac{1}{P}\int_{0}^{f_{max}}D\left(f,\theta \right)S\left(f\right)df.$$

**Figure 5:**
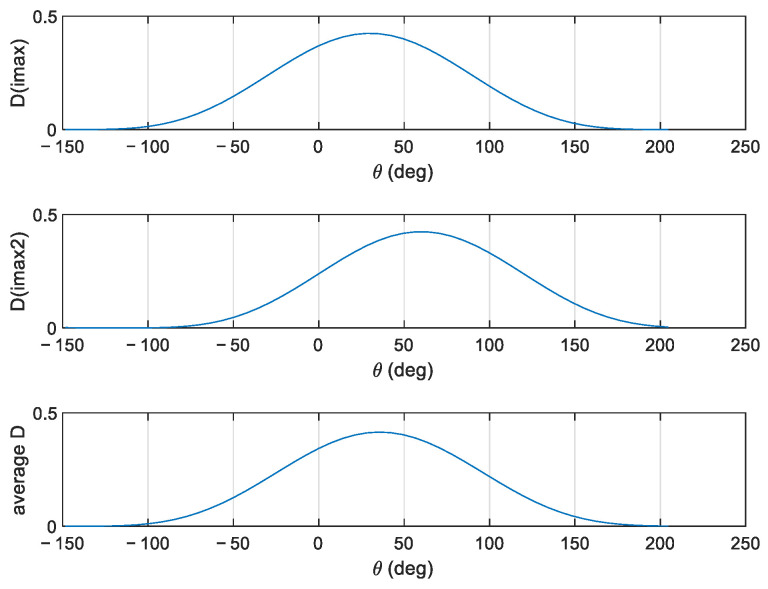
Directional spreading function at the two frequencies and overall function.

In Figure 5, the directional spreading functions at the two frequencies and the overall function are displayed.

The issue of spectral resolution has also been checked.

Towards this goal, two components of equal amplitude, H=1 m, were considered, at the same frequencies as in the example above. The former was fixed at $\theta_{01}=30{}^{\circ}$, and the latter was moved up to seeing two peaks in the overall directional spread function.

**Figure 6:**
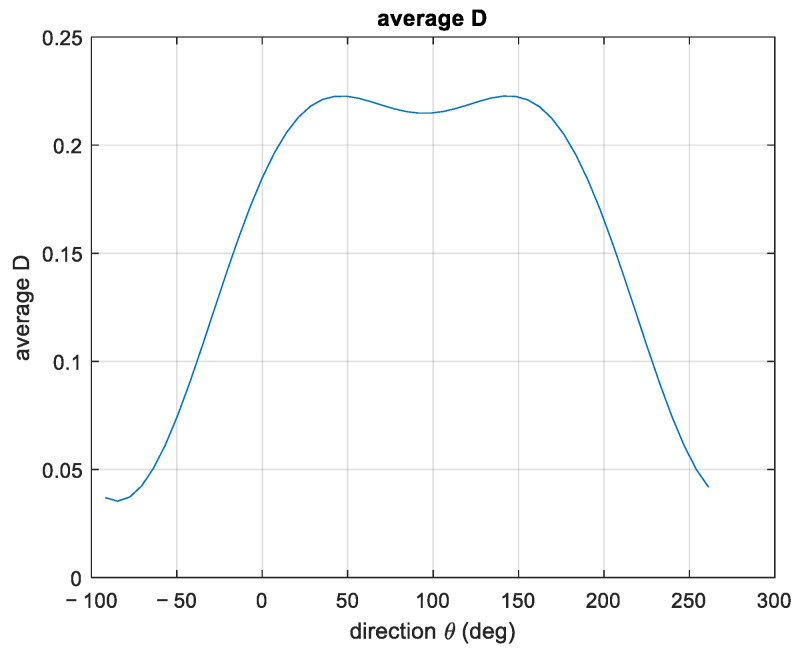
Overall directional spreading function, with ∆θ=130°.

This happened with ∆θ>120°. For example, in Figure 6, the case of ∆θ=130° is shown.

### 3.3. Testing on Continuous (Stochastic) Spectra

For this kind of testing, non-directional JONSWAP spectra, combined with a parametrical cosine-shaped DSF, were considered.

As is well known, the JONSWAP power spectral density (PSD) is defined by [29,33]

(24) $$S_{J}\left(f\right)=A_{\gamma }S_{PM}\left(f\right)\gamma^{exp\left(-0.5{\left(\frac{f-f_{p}}{{\sigma f}_{p}}\right)}^{2}\right)},$$

having been denoted by f the wave frequency, $A_{\gamma }=1-0.287ln\gamma$ the normalizing factor, $T_{p}$ the peak period, $f_{p}=\frac{1}{T_{p}}$ the peak frequency, σ the spectral width parameter, equal to 0.07 if $f\leq f_{p}$ and 0.09 otherwise, γ the peak enhancement factor, and $S_{PM}$ the Pierson–Moskowitz spectrum:

(25) $$S_{PM}\left(f\right)=\frac{5}{16}H_{s}^{2}f_{p}^{4}f^{-5}exp\left(-\frac{5}{4}{\left(\frac{f}{f_{p}}\right)}^{-4}\right).$$

For γ ranging from 1 to 7 and unimodal wave spectra, the peak period and the average period, $T_{m}$, are linked by the approximate formula

(26) $$\frac{T_{m}}{T_{p}}=a+b\gamma +c\gamma^{2}+d\gamma^{3}$$

where a = 0.7303, b = 0.04936, c = −0.006556, and d = 0.000361.

For the DSF, the following expression was used [27,34]:

(27) $$D\left(\theta \right)=\frac{1}{\Lambda \left(p\right)}\cos^{2p}\left(\theta -\alpha \right)$$

where $\Lambda \left(p\right)$ is a scaling factor that ensures that the function has a unitary integral, according to (4), α is the propagation direction, D is non-null only for $\theta \in \left[\alpha -\frac{\pi }{2},\alpha +\frac{\pi }{2}\right]$, and p governs the degree of spread, in that the higher p is, the lower the spread results.

Then, according to (1), the directional spectrum is

(28) $$S\left(f,\theta \right)=S_{J}\left(f\right)D\left(\theta \right).$$

**Figure 7:**
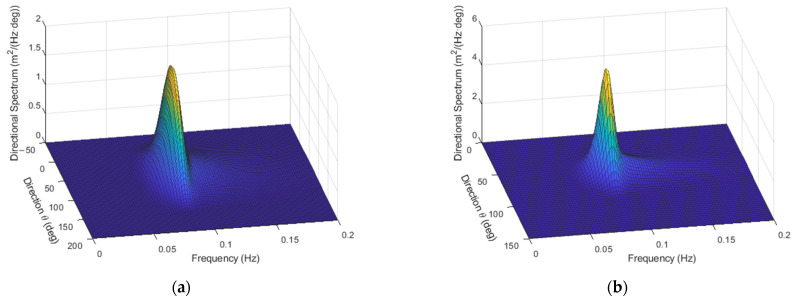
Directional spectra with p=1 (**a**) and p=10 (**b**), respectively.

As an example, with H=1 m, $T_{m}=10\;s$, $f_{p}=0.09\;Hz$, γ=6, and α=60°, with either p=1 or p=10, the directional spectra appear as in Figure 7.

The synthesis of an associated sea state field, which constitutes a realization of the stochastic field characterized by (28), can be obtained in different ways, including the double summation approach [35,36], which can be expressed in the following way:

(29) $$z\left(x,y,t\right)=\sum_{i=1}^{L}\sum_{j=1}^{M}a_{ij}\cos\left[k_{i}\left(x\cos\theta_{j}+y\sin\theta_{j}\right)-2\pi f_{i}+\varphi_{ij}\right]$$

with a slight abuse of notation, since here x and y denote (constant) sea surface spatial coordinates, while in (18) and (19), $x\left(t\right)$ and $y\left(t\right)$ denote (time-dependent) buoy signals. According to the random phase (RP) approach, the parameters in (29) can be specified as follows:

- $f_{i}=i∆f$
- $k_{i}={\left(2\pi f_{i}\right)}^{2}/g$
- $\theta_{j}=\theta_{0}+j∆\theta$
- $a_{ij}=\sqrt{2S\left(f_{i},\theta_{j}\right)∆f∆\theta }$
- $\varphi_{ij}=2\pi U\left[0,1\right]$

where U denotes a uniform probability distribution. For wave numbers $k_{i}$, a deep-water approximation has been applied. This is justified by the fact that the buoy to be considered in the case studies in Section 4 is deployed a few miles from the coast, at a water depth of 85 m. Such values are like those of the other RON buoys. This approach has been used extensively for directional wave simulation, although criticism has been raised against it, since it was noted that the resultant wave field is neither ergodic nor spatially homogeneous for finite values of L and M [36]. Yet, spatial homogeneity is no concern here, since a single observation point (the buoy) is considered. Indeed, limited ergodicity may be more critical; yet, since the estimation methods considered here have a relatively low spatial resolution, this approach has still been considered appropriate for the present testing and selected for its simplicity, leaving the exploration of different approaches to a future study devoted to high-resolution analyzers, as will be mentioned in the concluding section.

**Figure 8:**
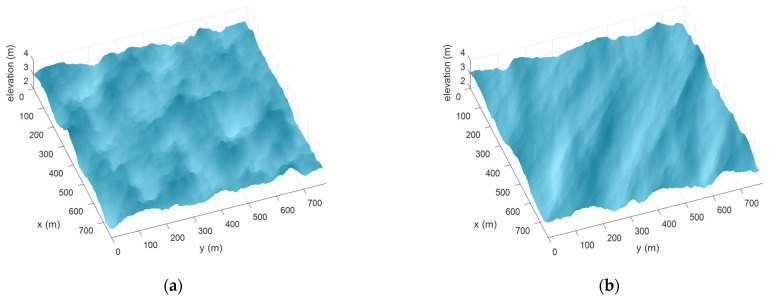
Realizations of the directional spectra reported in Figure 7, with p=1 (**a**) and p=10 (**b**), respectively.

Examples of synthesis of sea waves associated with spectra in Figure 7 are shown in Figure 8.

**Figure 9:**
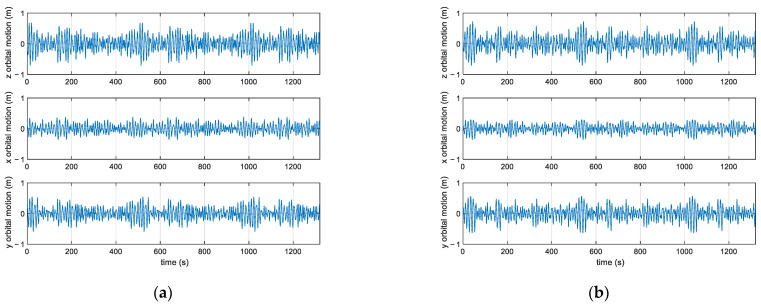
An example of simulated buoy signals, with p=1 (**a**) and p=10 (**b**).

The corresponding buoy signals, at spatial coordinates x=0 and y=0, are as follows:

(30) $$z\left(t\right)=\sum_{i=1}^{L}\sum_{j=1}^{M}a_{ij}\cos\left[-2\pi f_{i}+\varphi_{ij}\right]$$

(31) $$x\left(t\right)=\sum_{i=1}^{L}\sum_{j=1}^{M}{-a}_{ij}\cos\theta_{j}\sin\left[-2\pi f_{i}+\varphi_{ij}\right]$$

(32) $$y\left(t\right)=\sum_{i=1}^{L}\sum_{j=1}^{M}{-a}_{ij}\sin\theta_{j}\sin\left[-2\pi f_{i}+\varphi_{ij}\right]$$

to be compared to (17)–(19). For the simulation, parameters like those occurring in typical monitoring systems, i.e., total observation time: T=1320 s=22 min, sampling frequency: $f_{s}=2\;Hz$, were assumed. An example of such signals is presented in Figure 9.

**Figure 10:**
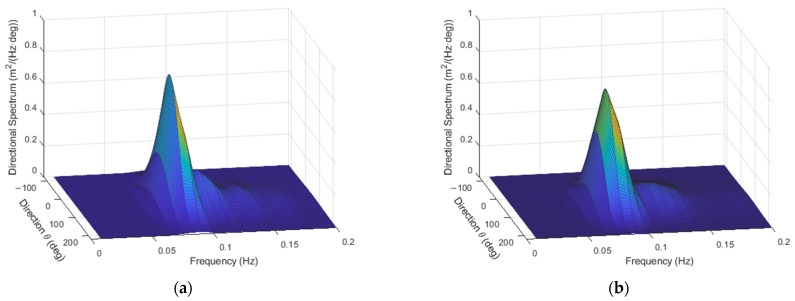
Estimated spectra, to be compared to those presented in Figure 7, with p=1 (**a**) and p=10 (**b**).

The corresponding estimated directional spectra, obtained through the WOSA method and through Equations (9)–(14), are reported in Figure 10.

From the testing, it appeared that the following is true:

- with medium-high values of p, e.g., p=10, the estimation of the propagation direction is more accurate, typically ±2°, while with small values of p, e.g., p=1, the uncertainty is of the order of ±5°;
- on the other hand, if the estimation of the directional spread is of interest, this will be worse with high values of p due to the convolution effect with the directional observation window; to enhance this, it would be necessary to consider other spectrum estimators, which is out of the scope of this study, since here the estimation of the direction is of primary interest.

To sum up, this testing campaign has confirmed the soundness of the method and provided insight for its conscious application.

## 4. Case Studies

### 4.1. The Site and the Instrumentation

The above procedures were applied to three test cases, representative of typical Mediterranean Sea state conditions, along Italian coasts west to Sardinia Island. In fact, they include

1. a mid-intensity storm with a prevailing Libeccio (a strong south-westerly wind blowing on the sea to the west of Italy) propagation direction;
2. a lower-intensity storm with a bimodal spectrum; and
3. a mid–high-intensity event, associated with a mistral wind (a strong, cold, dry wind that blows south through France to the Mediterranean).

**Figure 11:**
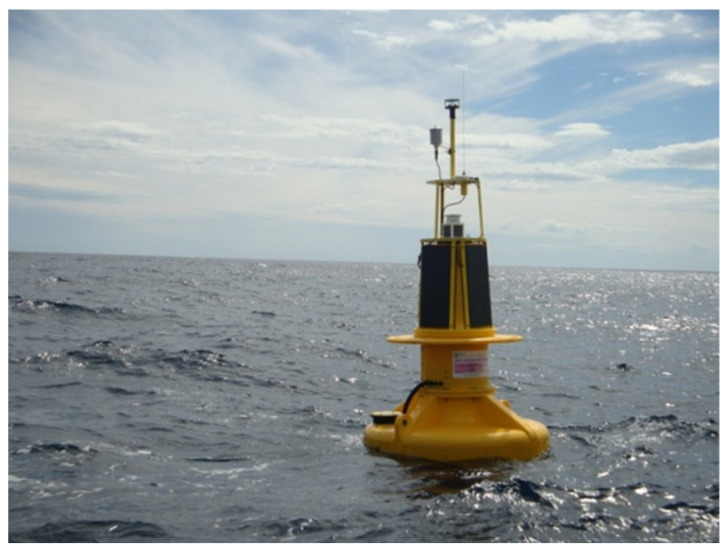
The Alghero buoy, April 2014.

**Figure 12:**
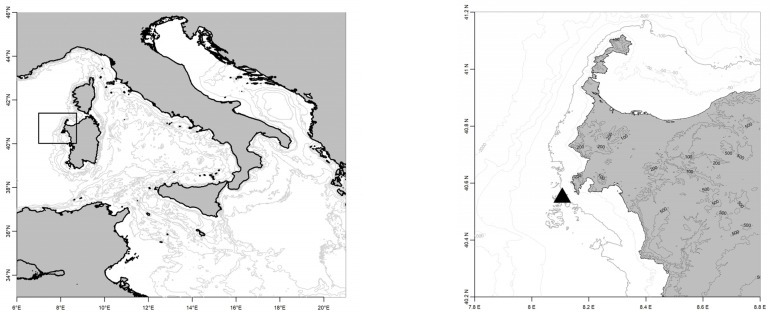
The Alghero buoy position.

Observations were taken from a RON buoy (model Axys Watchkeeper 1.7 m, depicted in Figure 11) equipped with the TRIAXYS wave sensor. The buoy is deployed in the Western Mediterranean Sea at 40°32′55′′ N–08°06′25′′ E, approximately 2.7 NM off the coast of Alghero (island of Sardinia, Italy) in 85 m of water, as in Figure 12. This met-ocean buoy (for joint monitoring of meteorological and oceanographic parameters) has a yellow polyethylene hull specially designed for installations in coastal areas, uses a powerful and energy-efficient controller to integrate signals from sensor systems and provides onboard directional wave data processing. It is widely used internationally and can be considered one of the reference tools for coastal sea monitoring networks.

### 4.2. Test Case 1: A Mid-Intensity Storm with a Libeccio Wind Propagation Direction

The first case event considered consists of a mid-intensity storm between 4 April and 6 April 2014. The significant wave height ($H_{m0}$) reached values of 3.71 m, which lies above the 95th percentile of the historical distribution. The whole time series of the significant wave height frequencies for the Alghero buoy presents a graph with an absolute maximum in the direction of the Mistral wind (approx. 310° N) and a secondary peak in the direction of the Libeccio wind (approx. 240° N).

Let us discuss the estimation of the directional spectrum in some detail. Available data include records of elevation, z, displacement toward the East, x, and displacement toward the North, y, of duration T=22.5 min, acquired each half hour. In [29], values of $T_{0}$ between 80 s and 120 s were found to perform best for this kind of data. Here, $T_{0}=120$ s was adopted, which corresponds to an apparent spectral resolution ∆f=8 mHz, an effective bandwidth $∆f_{e}=12\;mHz$, and a relative standard uncertainty (related to the variance of the estimate), $u_{rel}=0.23$. This has been considered as an acceptable trade-off between the conflicting requirements of having a good spectral resolution, which implies a low effective bandwidth, and a limited variance of the estimate.

**Figure 13:**
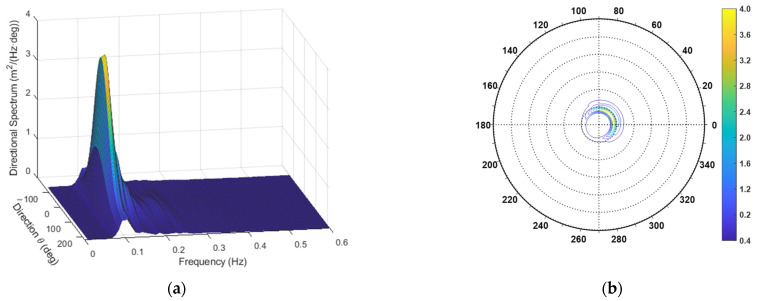
The directional spectrum for the first case study. (**a**) Three-dimensional plot; (**b**) Contour polar plot.

**Figure 14:**
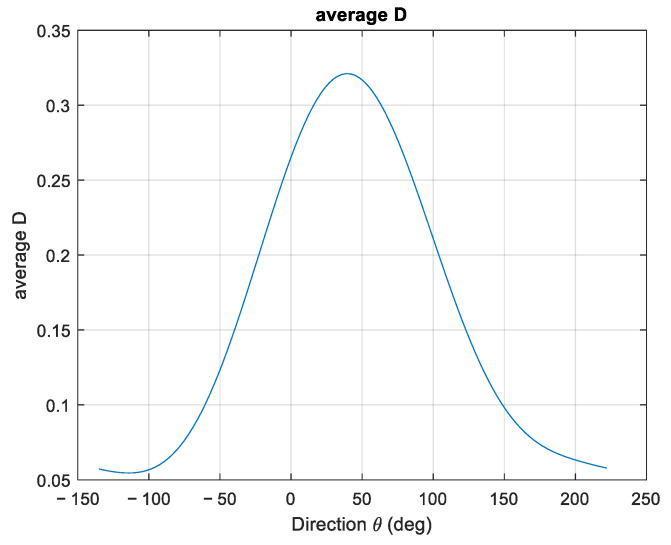
Overall direction spread function for the case study.

After obtaining the required spectra with this approach, the directional spectra were estimated through both the TFS and WTFS approaches. An example of one such spectrum is shown in Figure 13. The propagation direction was $\theta_{0}=46{}^{\circ}$, and the overall direction spread function appeared as in Figure 14.

**Figure 15:**
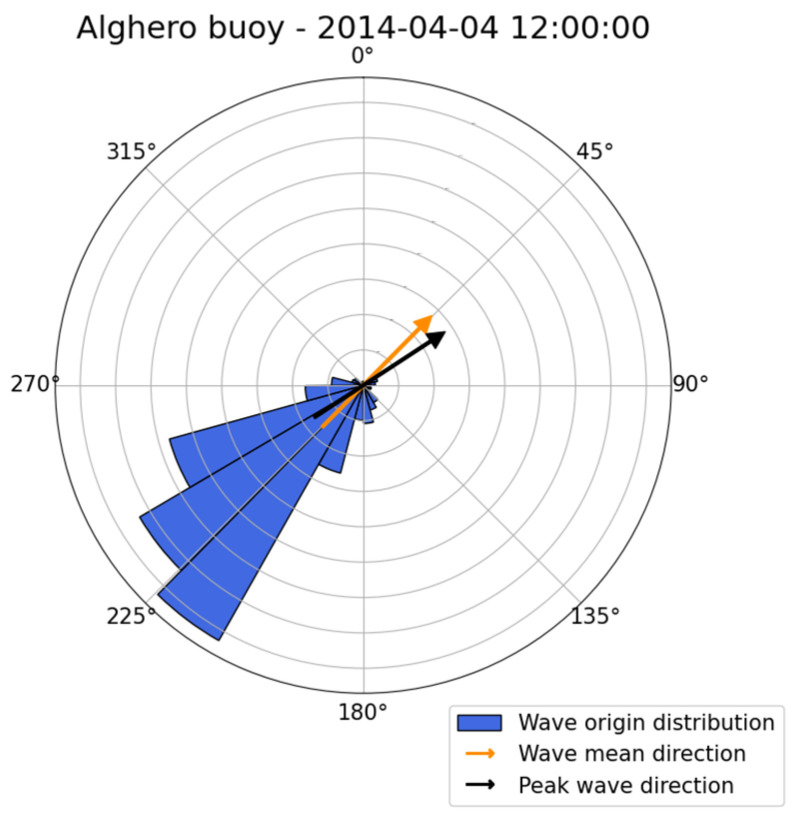
Wave origin direction distribution integrated over one run, for case study 1. Wave weighted mean direction (orange arrow) and wave peak direction (black arrow) are also reported.

Considering the overall monitoring of the meteorological event, in Figure 15, an example of the directionality analysis output on the Alghero buoy is reported. The run used is 4 April 2014, 12:00 UTC. The direction reported in the polar histogram refers to the origin of the wave and is weighted by the $C_{ZZ}$ spectrum. The weighted average direction and the peak direction are reported as well. Here, the NDBC (National Data Buoy Center) convention is used, where direction is measured clockwise from true north [16].

The distribution in Figure 15 shows a clear prominence of southwest wave origin, compatible with a strong Libeccio component.

### 4.3. Test Case 2: A Lower Intensity Storm with a Bimodal Spectrum and Libeccio Wind Direction

The second case event considered lies within a moderate sea state event on 22 February 2010, with a significant wave height ($H_{m0}$) around 1.5 m. The event features a dominant Libeccio component, around 210° N. This meteorological event follows a stronger storm with peak intensity on 20 February ($H_{m0}\sim 6\;m$), which is instead dominated by a prominent Mistral component.

**Figure 16:**
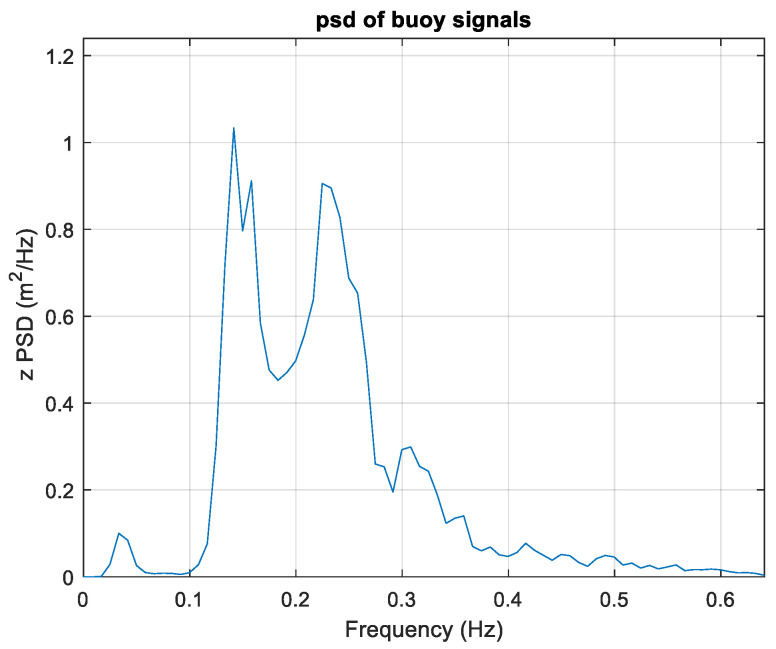
The non-directional (heave) spectrum for case study 2.

**Figure 17:**
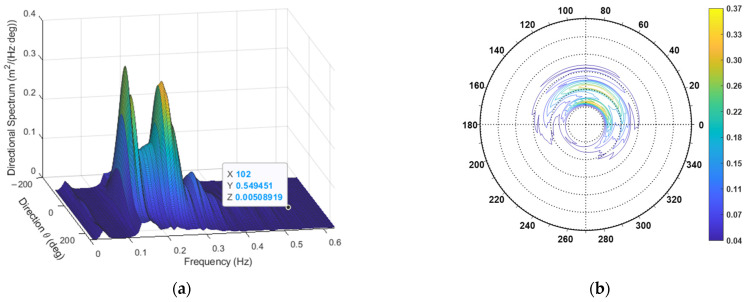
The directional spectrum for the second case study. (**a**) Three-dimensional plot; (**b**) Contour polar plot.

Concerning spectrum estimation, the observation conditions were similar to those in the previous case; therefore, the same estimation parameters were used, with, in particular, $T_{0}=120$ s. Both the TFS and WTFS approaches were applied. The non-directional (heave) spectrum is reported in Figure 16, and the directional one, obtained by the WTFS approach, is displayed in Figure 17.

**Figure 18:**
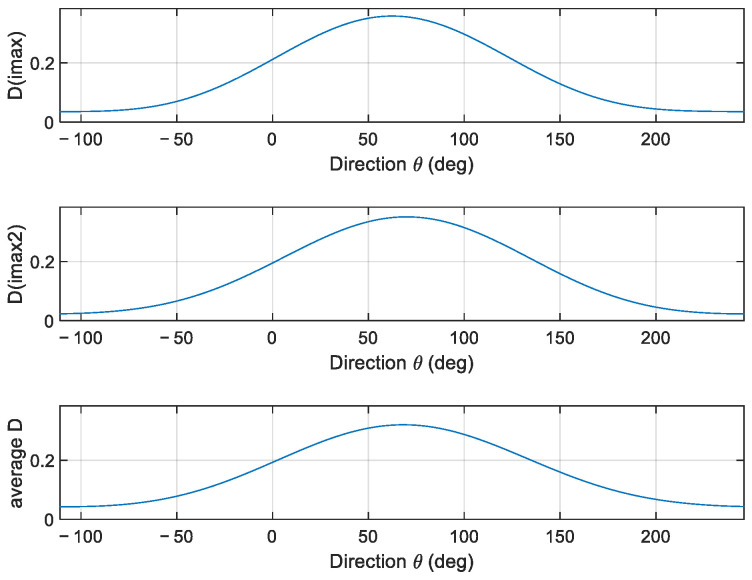
The directional spread functions at peak frequencies and the overall (average) one.

The spectrum exhibits two peaks at $f_{1}=0.14\;Hz$ and $f_{2}=0.22\;Hz$, respectively. However, the propagation distributions are very similar, being $\alpha_{1}=63{}^{\circ}$ and $\alpha_{2}=69{}^{\circ}$. The overall direction is α=68°, showing that there is more energy in the higher frequency region. The estimated DSF at peak frequency and the overall DSF are reported in Figure 18.

**Figure 19:**
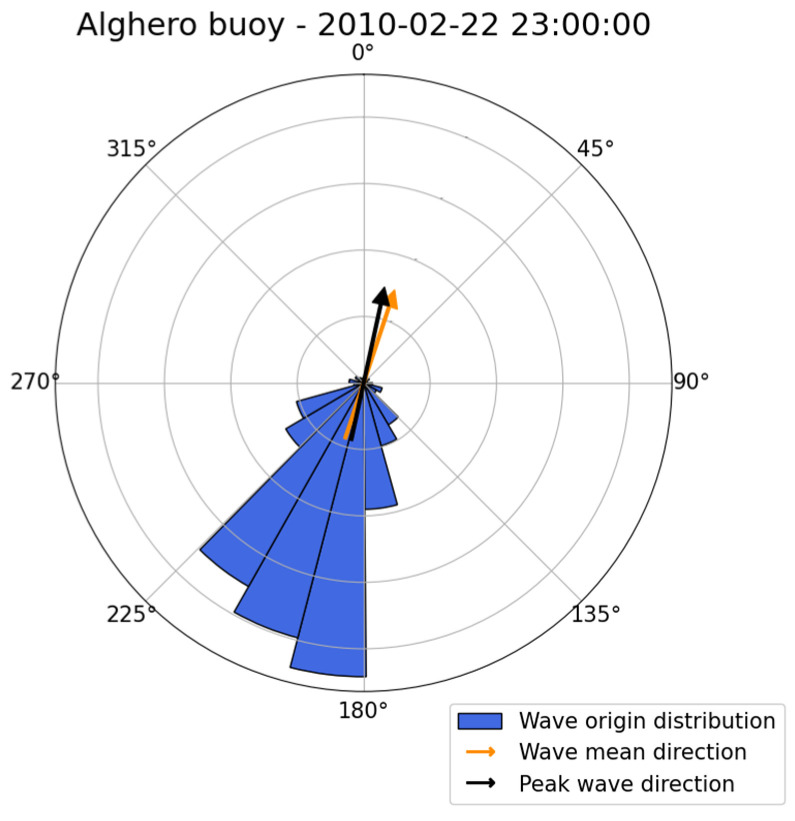
Wave origin direction distribution integrated over one run, for case study 2. Wave weighted mean direction (orange arrow) and wave peak direction (black arrow) are also reported.

Figure 19 summarizes the meteorological event in terms of its directional features. The run used is 22 February 2010, 23:00 UTC. The same convention used for Figure 15 is applied. The distribution reported in Figure 19 shows a clear Libeccio component in the southwest direction.

### 4.4. Test Case 3: A Mid–High-Intensity Event, Associated with a Mistral Wind

**Figure 20:**
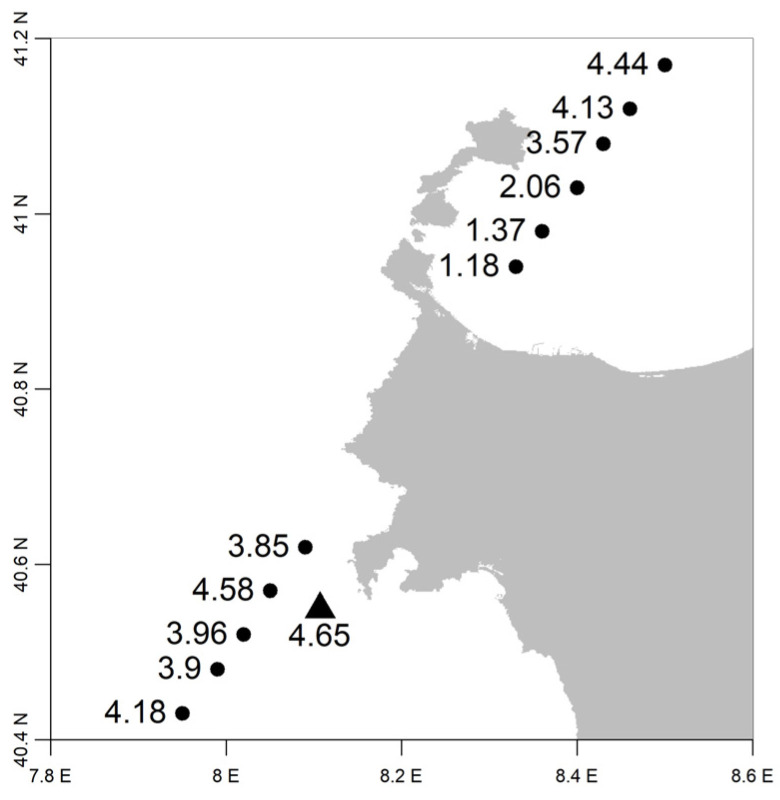
Significant wave heights (in meters) near the Alghero buoy on 20 February 2010; triangle: value observed by the buoy at 9:30 UTC; circles: values observed by satellite altimeter Jason-2 at 9:18 UTC.

The third case event analyzed in this work is situated within a storm that occurred within 19 February and 21 February 2010, with a significant wave height in the range of $\left(3-5\right)m$. The dominant wind component is the Mistral one, pointing to the northwest direction. For the validation of the method, in this case study, the significant wave height detected by the simultaneous observation of the Jason-2 satellite altimeter was verified, which is independent of the data observed at sea level from the buoys, as shown in Figure 20.

**Figure 21:**
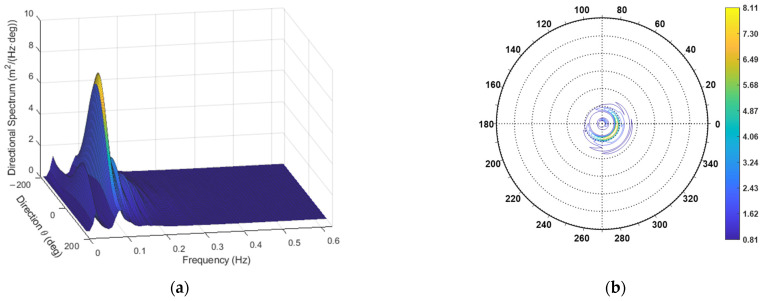
The directional spectrum for the third case study. (**a**) Three-dimensional plot; (**b**) Contour polar plot.

The estimation of the directional spectrum, still made with the observation window having a duration $T_{0}=120\;s$, has yielded the directional spectrum reported in Figure 21.

**Figure 22:**
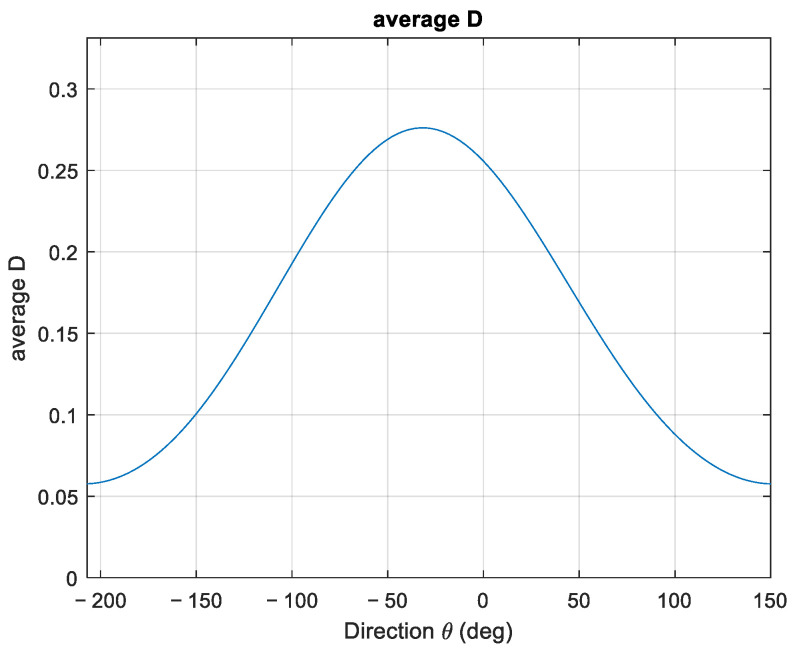
Average directional dispersion function for case study 3.

The corresponding average dispersion function is presented in Figure 22.

**Figure 23:**
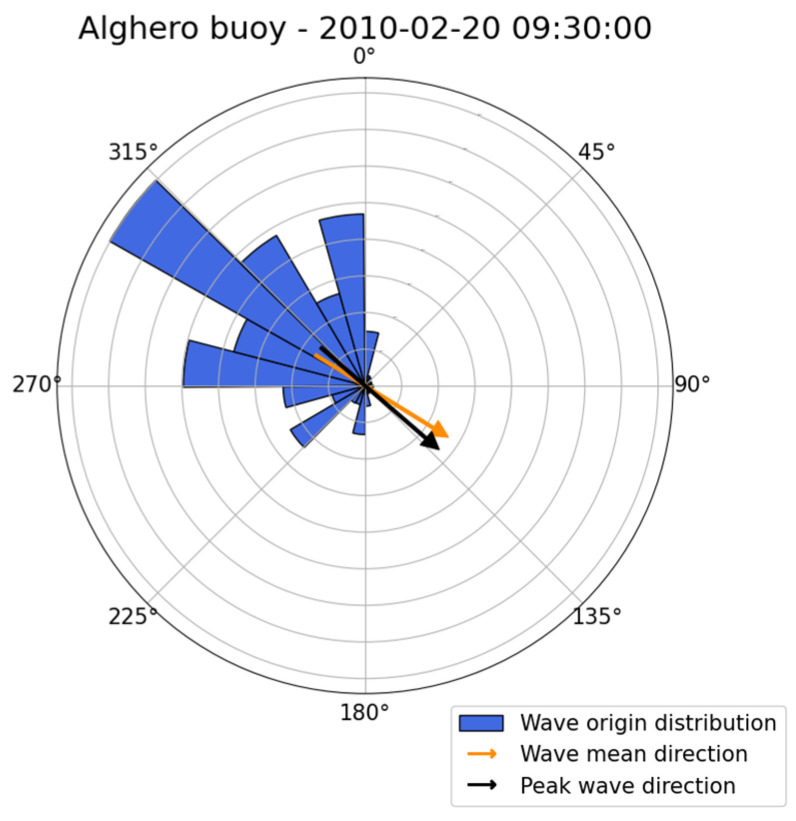
Wave origin direction distribution integrated over one run for case study 3. Wave weighted mean direction (orange arrow) and wave peak direction (black arrow) are also reported.

The run considered is 20 February 2010, 09:30 UTC, which features a value for $H_{m0}$ of 4.65 m. The outcome of the directionality analysis is reported in Figure 23, and the convention follows the same rules adopted for Figure 15 and Figure 19. The directional distribution confirms a strong northwest component.

## 5. Results and Comments

The estimation of directional spectra of sea has been investigated, considering the needs of a national (Italian) sea-waves monitoring system. Two main algorithms, namely the truncated Fourier series (TFS) and the weighted truncated Fourier series (WTFS), have been implemented, tailored to the output of the buoys under consideration, which is calculated in terms of three-dimensional displacements rather than on the more common heave, pitch, and roll system. The methods have been tested by both simulation and application to selected test cases.

Simulations have been performed both by discrete-spectrum signals and by signals generated by continuous spectra. The former study has shown the following aspects.

- Both methods correctly identify the propagation direction at each frequency in the spectrum.
- An average overall directional spreading function has been defined and calculated: such a function is useful in that it properly identifies the mean propagation direction of the overall process, but it has a limited directional resolution, since, in the (worst) case of components having the same significant elevation, it resolves the direction only if they are over 120° apart.
- Even if the TFS has a slightly higher directional resolution, it has the severe drawback of possibly exhibiting negative spectrum values. Therefore, although it seems widely used [16], the authors consider the WTFS approach generally preferable.
- For both methods, the outcome of the directional dispersion is severely affected by the limitation of the terms of the truncated Fourier series.

The simulation of signals generated by continuous spectra was performed by adopting a JONSWAP spectrum multiplied by a parametric cosine DSF, depending upon a parameter p the governs the spread, in that a high value of p corresponds to a low spread dispersion and vice versa. The signals were generated by the double summation random phase approach. Simulation highlighted the following aspects:

- The WTFS was still considered preferable for the same reasons discussed above.
- Concerning the estimation of the non-directional spectrum, the Welch method with a Hanning taper and a 50% segment overlap proved to be effective. The most important parameter to be chosen, then, is the local window duration, $T_{0}$. With the typical recording time of the buoy system under consideration, T=22.5 min, and the optimum choice was $T_{0}=120\;s$, which corresponds to an apparent spectral resolution ∆f=8 mHz, an effective bandwidth $∆f_{e}=12\;mHz$, and a relative standard uncertainty $u_{rel}=0.23$.
- The estimation of the direction is reliable. It was possible to make an evaluation of its uncertainty, ranging from ±2° for highly directional seas (p=10) to ±5° for dispersed sea conditions (p=1).
- On the other hand, the outcome of the dispersion spread is generally unsatisfactory, and the corresponding error is greater for highly directional seas, where this information is more important. This appeared to be the main drawback to be faced in future studies.

Then, the preferred method (WTFS) was tested with real signals recorded by a RON buoy (model Axys Watchkeeper) deployed in the Western Mediterranean off the coast of Alghero (Sardinia Island). Three test cases were considered, representative of typical Mediterranean Sea state conditions, along Italian coasts, with different wave intensities and wind directions, including both mono- and bimodal spectra. In this regard, some features of the Mediterranean Sea deserve attention. In fact, due to the reduced dimensions with respect to the oceans, the complex orography of the coasts and the existence of mountain ranges that often affect the local climate, and the presence of a great number of islands and open and enclosed or semi-enclosed basins, the physical processes appear accentuated or reduced [37,38]. It is well known that relevant events in terms of waves and currents are shorter and less severe with respect to the corresponding events in the oceans. On the other hand, storm surges are relevant in particular basins such as the northern Adriatic Sea. In this context, Mediterranean and ocean waves are different in terms of intensities and spectral parameters. In fact, in the case of relevant events, ocean wave spectra appear well concentrated around the modal value, while often the Mediterranean wave spectra appear noisier because, in general, more than one component is observed. Therefore, the testing based on real case studies was particularly significant for this study. In all three cases, the reliability of the method was confirmed by additional meteorological data, including, in one case, satellite observations.

The main drawback of the method is its limited directional resolution. Therefore, future studies will be devoted to investigation of alternative high-resolution methods, like the Maximum Entropy Method (MEM), used at the CDIP [18]. Yet, they may be less robust since they may produce artifacts [27,28]. Then, another possibility is to consider spectral partitioning, i.e., the process of identifying and separating different systems—typically wind and swell—that coexist in a complex sea state [39,40]. They could then be treated separately in terms of directional spectra.

## 6. Conclusions

The lines of development of the postprocessing of wave buoys= data for a national wave buoy monitoring system have been outlined by presenting a method that is independent from the buoy vendor encapsulated algorithms. The usage of such private algorithms prevents a government agency from easily switching vendors when the contract period expires, since it would require changing the output standards. In this regard, the estimation of the directional wave spectrum has been deeply considered, with respect to two key methods, namely TFS and WTFS. The methods have been implemented, and their characteristics have been first investigated through simulated test signals, including both discrete spectrum (deterministic) phenomena and then continuous spectrum (stochastic) processes. This investigation has provided useful insight for their practical application. The application to three case studies representative of typical sea states in the central Mediterranean Sea has also been considered, providing an additional validation opportunity. The following results emerged:

- The WFTS method is preferrable to the FTS one.
- The method provides a reliable estimation of the main wave propagation direction, whose uncertainty was estimated by simulation and whose reliability was confirmed by infield testing.
- The averaged directional dispersion function provided a useful overall description of the energy directional propagation.

The main limitation of the method consists of a limited directional resolution. Therefore, successive developments are envisioned, including the implementation and testing of other methods, with a special eye on directional resolution and the study of spectral partitioning for the automatic recognition of multimodal sea states and an accurate estimation of directions in such cases.

## Data Availability

Data equivalent to those used in simulation (not strictly equal, due to the stochastic nature of simulation) are available upon request to the authors. Experimental data, in an aggregated form, are available at https://dati.isprambiente.it (accessed on 29 May 2023).
